# Technological Advances in a Therapy of Primary Open-Angle Glaucoma: Insights into Current Nanotechnologies

**DOI:** 10.3390/jcm12185798

**Published:** 2023-09-06

**Authors:** Julita Zembala, Alicja Forma, Roksana Zembala, Jacek Januszewski, Patryk Zembala, Dominik Adamowicz, Grzegorz Teresiński, Grzegorz Buszewicz, Jolanta Flieger, Jacek Baj

**Affiliations:** 1 University Clinical Center, Medical University of Warsaw, Lindleya 4, 02-005 Warsaw, Poland; 2 Department of Forensic Medicine, Medical University of Lublin, Jaczewskiego 8b, 20-090 Lublin, Poland; grzegorz.teresinski@umlub.pl (G.T.); grzegorz.buszewicz@umlub.pl (G.B.); 3 Faculty of Medicine, Cardinal Stefan Wyszynski University in Warsaw, Wóycickiego 1/3, 01-938 Warsaw, Poland; roksana.zembala@gmail.com; 4 Department of Human Anatomy, Medical University of Lublin, Jaczewskiego 4, 20-090 Lublin, Poland; jacek.januszewski000@gmail.com (J.J.); jacek.baj@umlub.pl (J.B.); 5 Faculty of Medicine, Medical University of Warsaw, Banacha 1A, 02-097 Warsaw, Poland; patrykx0@hotmail.com; 6 University Clinical Center, Medical University of Warsaw, Banacha 1A, 02-097 Warsaw, Poland; dominik.adamowicz91@gmail.com; 7 Department of Analytical Chemistry, Medical University of Lublin, Chodźki 4A, 20-093 Lublin, Poland; jolanta.flieger@umlub.pl

**Keywords:** open-angle glaucoma, treatment, advanced technology, ophthalmology, hydrogel, liposomes, contact lenses, niosomes, nanoemulsion, nanocrystal

## Abstract

Glaucoma is a leading cause of irreversible blindness and is characterized by increased intraocular pressure (IOP) and progressive optic nerve damage. The current therapeutic approaches for glaucoma management, such as eye drops and oral medications, face challenges including poor bioavailability, low patient compliance, and limited efficacy. In recent years, nanotechnology has emerged as a promising approach to overcome these limitations and revolutionize glaucoma treatment. In this narrative review, we present an overview of the novel nanotechnologies employed in the treatment of primary open-angle glaucoma. Various nanosystems, including liposomes, niosomes, nanoparticles, and other nanostructured carriers, have been developed to enhance the delivery and bioavailability of antiglaucoma drugs. They offer advantages such as a high drug loading capacity, sustained release, improved corneal permeability, and targeted drug delivery to the ocular tissues. The application of nanotechnologies in glaucoma treatment represents a transformative approach that addresses the limitations of conventional therapies. However, further research is needed to optimize the formulations, evaluate long-term safety, and implement these nanotechnologies into clinical practice. With continued advancements in nanotechnology, the future holds great potential for improving the management and outcomes of glaucoma, ultimately preserving vision and improving the lives of millions affected by this debilitating disease.

## 1. Introduction

Glaucoma is a chronic disease that damages the optic nerve and leads to a progressive loss of retinal nerve fibers, which form the optic nerve responsible for transmitting visual information from the retina to the brain [1]. As a result, it causes visual impairment and blindness that initially affects the peripheral visual field and gradually progresses to involve central vision [1,2]. Glaucoma is the second-leading cause of irreversible blindness worldwide, with preventability emphasized the World Health Organization (WHO) in 80% of cases [3]. Early diagnosis is challenging due to its diverse forms and lack of symptoms. By the time patients notice a deterioration in their vision and seek medical attention, they may have already lost up to 40% of their retinal ganglion cell axons. Primary open-angle glaucoma, which accounts for up to 90% of cases and has a global prevalence of 3.1%, is six times more frequent than primary angle closure glaucoma [3,4]. This is the focus of our review.

**Figure 1:**
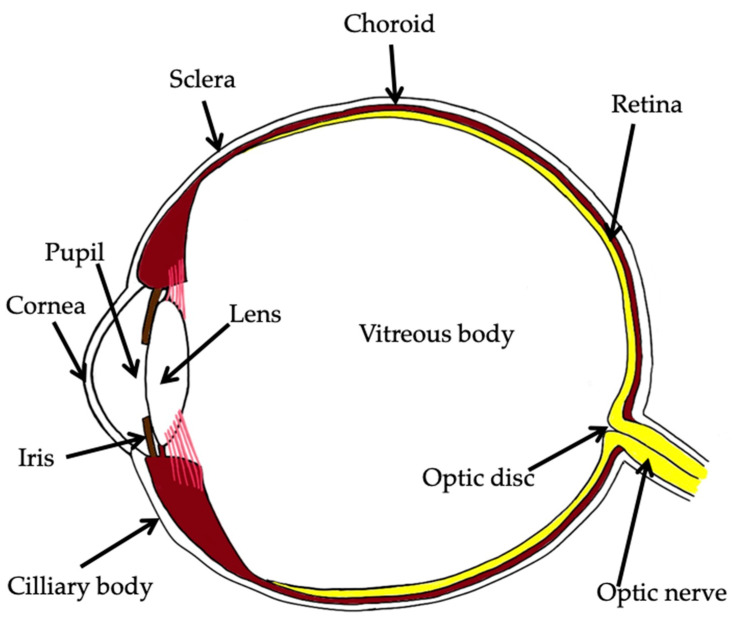
Schematic of the eye showing its essential elements.

The main modifiable risk factor in glaucoma is intraocular pressure [1]. Reducing IOP by 30–50% from the baseline can effectively slow down the progression of glaucoma [1]. The eye has anterior and posterior chambers filled with aqueous humor, which is produced by the ciliary body epithelium and circulates from the posterior chamber to the anterior chamber through the pupil (Figure 1) [2].

The aqueous humor nourishes the lens and cornea. Proper drainage from the anterior chamber is crucial for maintaining normal IOP. Insufficient drainage leads to an increase in IOP [1,5,6,7]. While the normal range for IOP is 10 to 21 mmHg, not all glaucoma patients exhibit abnormal IOP values, although most have an IOP higher than 21 mmHg [1].

Various factors contribute to the pathogenesis of glaucoma, such as advanced age, individuals of African, Asian, or Hispanic descent, and mechanical, ischemic, and oxidative factors [1,5]. IOP results in compression of the lamina cribrosa, disrupting the flow of axoplasm between the cell body and synapse and causing apoptosis [8,9]. The elevated intraocular pressure leads to displacement of the lamina cribrosa, compressing blood vessels and optic nerve fibers, which can cause ischemia and neuronal cell death [8,9]. Oxidative stress contributes to increased IOP by impeding the outflow of the aqueous humor [6]. 

Glaucoma can be classified into primary, secondary, and congenital types [2,5,8]. Primary open-angle glaucoma is the most common form of glaucoma, accounting for 90% of cases [4]. It is caused by a disturbance in the normal drainage of the aqueous humor, leading to its impaired outflow and subsequent elevation of intraocular pressure [2,3]. Acute angle-closure glaucoma occurs when the iridocorneal angle closes, elevating IOP. Secondary glaucoma is linked to various eye and systemic conditions, surgeries, and medications [9,10,11,12]. Congenital glaucoma occurs in newborns due to developmental issues with aqueous humor drainage during prenatal stages [5].

### Potential Treatment Strategies

The main goal of treatment is to delay the progression of neuropathy and prevent total vision loss. However, the nerve fibers that have already been damaged cannot be restored. Pharmacological treatment aimed at lowering intraocular pressure can significantly slow down the process of vision loss [13].

Surgical treatment includes various approaches to enhance the drainage of the aqueous humor through the Schlemm’s canal or to reduce its production [2,3,14]. Standard procedures include glaucoma filtering surgeries with mitomycin C, glaucoma drainage devices, and laser therapy [2,14]. However, there is a group of patients who are unable or unwilling to undergo surgery; therefore, pharmacotherapy, particularly the use of intraocular pressure-lowering medications, is usually employed. According to the American Academy of Ophthalmology Preferred Practice Pattern (2020), the guidelines recommend that an initial reduction in IOP by 20–30% is an appropriate goal to slow down the progression of glaucoma, even in cases of normal tension glaucoma [15].

Multiple categories of topical medications are available for reducing intraocular pressure. Among these, prostaglandins are considered the initial choice for medical treatment [16,17]. Prostaglandins exert their effects by binding to prostaglandin receptors within the eye, thereby enhancing the outflow of the aqueous humor. They specifically promote the drainage of fluid through the uveoscleral pathway, which involves the ciliary body and the base of the iris. Commonly prescribed medications in this class include latanoprost, bimatoprost, travoprost, and tafluprost.

An alternative to prostaglandins for the treatment of glaucoma is the use of β-adrenergic blockers, such as timolol and betaxolol [17,18]. These medications reduce the production of aqueous humor in the eye. While these medications do not have many contraindications, their lack of selectivity leads to side effects, making them completely contraindicated in patients with asthma, chronic obstructive pulmonary disease (COPD), or heart failure.

Timolol in particular is widely used as a first line treatment for glaucoma [18]. However, its effectiveness can be enhanced when used in combination with prostaglandin analogs, which are known to be more effective in reducing intraocular pressure [17]. Another beta blocker, betaxolol, is a selective beta blocker that specifically binds to β1 receptors, resulting in reduced systemic side effects. However, it is not as potent as timolol in lowering intraocular pressure.

Another group of drugs includes carbonic anhydrase inhibitors, which are involved in the active secretion of aqueous humor [17]. When this enzyme is inhibited, the production of bicarbonate is reduced and ion secretion is altered, leading to a decrease in aqueous humor production and, consequently, a reduction in intraocular pressure [19]. Initially, oral CAIs such as acetazolamide and methazolamide were used. However, these have systemic hypotensive effects, cause many side effects, and have been largely replaced by topical CAIs such as brinzolamide and dorzolamide [16]. 

α adrenergic agonists are sympathomimetic drugs that stimulate alpha adrenergic receptors, inhibiting adenylate cyclase and reducing intracellular cAMP [17,19]. As a result, the production of aqueous humor in the ciliary body decreases. Examples of such drugs include brimonidine and dipivefrin, which exhibit both alpha and beta agonist activity, as well as clonidine and apraclonidine, which specifically act as alpha agonists.

Apraclonidine is primarily used as short-term adjunctive therapy following laser treatment or when the current therapy fails to sufficiently lower intraocular pressure [20]. However, it may cause drowsiness and orthostatic hypotension as side effects. In contrast, brimonidine is used as a monotherapy in cases where beta blockers are contraindicated or in combination with them to achieve the desired intraocular pressure levels [20]. 

Miotics such as pilocarpine have been widely used for a long time and are effective in reducing intraocular pressure by enhancing the transtrabecular outflow [17,19]. The local side effects of miotics include involuntary accommodation, which can be bothersome in patients under the age of 45, and pupillary constriction, which is particularly inconvenient during nighttime and can decrease visual acuity in eyes with cataracts. However, pupillary constriction can also increase the depth of focus due to the stenopeic effect. This makes miotics beneficial in eyes with artificial intraocular lenses following cataract surgery. Importantly, miotics do not cause significant systemic side effects.

## 2. Objectives of the Review and Search Strategy

The objective of this paper was to conduct a narrative review of the available literature regarding novel technologies for the treatment of primary open-angle glaucoma. The search of the literature was performed using of three databases—PubMed, Web of Science, and Scopus until May 2023. The initial search strategy was as follows: (glaucoma) AND (nanotechnology OR nanomedicine). After obtaining 129,819 results from the years 1961 to 2023, we reviewed the articles in terms of their novelties regarding glaucoma treatment, then focused on the last 10 years. After the first identification, amongst the 129,819 articles, the ones associated with glaucoma and new nanotechnologies were chosen. The second identification of the articles included the following search string: (nanotechnology OR nanomedicine) AND (glaucoma) AND (inserts OR contact lenses OR penetration enhancers OR hydrogels OR liposomes OR niosomes OR nanoemulsions OR nanosuspensions OR nanocrystals OR nanomicelles OR polymeric nanoparticles OR solid lipid nanoparticles OR nanostructured lipid carriers OR dendrimers OR cubosomes OR olaminosomes OR bilosomes). A total of 103 articles were found to be associated with the topic of this study, and these were included in the qualitative synthesis. The authors chose only the articles published in English.

## 3. Drug Penetration through the Ocular Surface

Despite the effectiveness of medical therapies for glaucoma, the current delivery system, primarily through eye drops, has several limitations and drawbacks. One of the main challenges is the frequency of administration, with eye drops often needing to be applied one to three times per day [21]. This regimen can lead to suboptimal use and a lack of adherence to the prescribed treatment, especially among the elderly population, which is more commonly affected by glaucoma. 

Studies have shown that a significant proportion of patients encounter difficulties or express concerns with the administration of their glaucoma medications, resulting in reduced adherence to long-term therapy [22]. This highlights the need for alternative delivery methods that improve patient compliance. 

Moreover, after the application of eye drops, the bioavailability of the medication in the anterior chamber of the eye is relatively low [23]. Animal studies have demonstrated that the typical aqueous humor bioavailability of a drug following eye drop administration ranges from only 1% to 10% [23,24]. This limited bioavailability is attributed to various physiological barriers that impede the penetration of drugs into the eye. 

Additionally, ocular surface diseases, including dry eye disease, conjunctivitis, blepharitis, and other inflammatory or infectious diseases can further complicate drug penetration [25]. They can disrupt the integrity of the corneal and conjunctival barriers, affecting drug absorption and distribution by altering the tear film composition, decreasing the precorneal residence time, and altering corneal permeability. 

These limitations pose a challenge in achieving optimal drug delivery to the targeted tissues within the eye and may contribute to the variability in treatment response among patients [26]. Overcoming these barriers and improving the bioavailability of glaucoma medications in the anterior chamber are important considerations for the development of more efficient and effective delivery systems.

## 4. Major Routes for Drug Delivery

The eye has natural defenses that provide high resistance to the penetration of active substances, including drugs, through its surface [2,23,26]. The eye can be anatomically divided into two parts: the anterior and posterior segments. The anterior segment includes tears, the cornea, conjunctiva, aqueous humor, anterior and posterior chamber, iris, pupil, ciliary body, and lens. The posterior segment consists of the vitreous body, sclera, sclera, choroid, retina, Bruch’s membrane, retinal blood vessels, and optic nerve. The various parts of the eye create obstacles to drug absorption, such as tears, naso-lacrimal drainage, and blinking. Drug installations stimulate reflex tearing and become diluted within the tear film, resulting in a reduction in the bioavailability [27]. The blood–ocular barrier is responsible for the regulation of IOP, aqueous humor circulation and composition, tear dilution, and turnover. Mucin is an ingredient of the tear film and forms a permeation barrier, restricting topically administered drugs due to its hydrophilic activity. Tear drainage, dilution in the tear film, and the mucin layer are the main limitations to topically applied ophthalmic medications.

Various layers of the conjunctiva, cornea, and sclera limit drug absorption through topical administration. The cornea is a protective structure at the front of the eye made of several different layers [28,29,30,31]. It can be divided into the epithelium, stroma, and endothelium. The epithelial layer is composed of total keratinized cells functioning as a mechanical barrier for hydrophilic drugs. Four different types of tight intercellular connections keep the corneal epithelium attached and preserve its barrier function [29,30]. Tight junctions (zonula occludens) are focal connections between nearby cells that function as a barrier to the diffusion of drugs in the first apical layer of the epithelium. The next type is Bowman’s layer, which due to desmosomes and tight junctions acts as a defense against microbial factors. The stroma, consisting of connective tissue and extracellular matrix, constitutes approximately 90% of the corneal thickness and has hydrophilic properties [30]. Below the stroma, there are the Descemet’s membrane and the endothelium, which separate the stroma and aqueous humor, helping to maintain selective carrier-mediated transport.

The cornea serves as a critical barrier for the transport of active substances through the eye. The intracellular route is the primary pathway for drugs, facilitating the diffusion of small lipophilic substances through the epithelium and endothelium [32]. On the other hand, hydrophilic molecules with a hydroxyl group use the extracellular pathway between the epithelial cells for transport [33]. The corneal epithelium, stroma, and endothelium contribute to the permeation of drug molecules into the anterior chamber, where they exert their pharmacological actions. For instance, drugs have the ability to bind to melanin in the ciliary body and iris [30]. They can also bind to the plasma proteins, extending their duration [34]. The remaining drug and metabolites are eliminated through the conventional route via the trabecular meshwork and Schlemm’s canal into the systemic bloodstream, or through the unconventional uveoscleral route [34,35]. The uveoscleral route involves drainage of the aqueous humor and/or drugs and their metabolites through the ciliary muscle and supraciliary and suprachoroidal spaces, either entering the choroid and draining through the vortex veins or passing across the sclera to be absorbed by orbital vessels. 

In addition, a route called the “uveolymphatic pathway” involves the drainage of aqueous humor through lymphatic channels in the human ciliary body [36]. This pathway may offer a new target for glaucoma treatment. Only a small portion of the drug penetrates the iris and diffuses via aqueous humor flow. The drug concentrations in the vitreous are much lower compared to the aqueous humor and cornea [32].

The conjunctiva serve as an alternative pathway for drug absorption into the eye. Unlike the cornea, the conjunctiva have larger intercellular spaces, providing better permeability for hydrophilic substances [33]. Active substances can be transported through the ocular surface layers via various routes, including the intracellular and extracellular pathways, endocytosis, the subconjunctival space (through injections and iontophoresis), or through active transport mechanisms [37]. 

Absorption through the conjunctiva is particularly advantageous for drugs with low corneal permeability, such as proteins and large molecules [37,38]. Hydrophilic drugs, proteins, and large molecules can penetrate through the conjunctiva and sclera. Although drugs administered via the non-corneal route can reach the vitreous cavity through passive diffusion, their concentration in the anterior chamber is approximately 20 times lower compared to the corneal route [33]. It is worth noting that the conjunctiva and underlying sclera exhibit higher permeability and offer a larger surface area for absorption into the bloodstream compared to the cornea [33]. 

Furthermore, certain topical medications used for lowering intraocular pressure, such as timolol and betaxolol, have been observed to accumulate in structures like Tenon’s capsule, periocular tissues, and the sclera in both normal cynomolgus monkeys and glaucomatous patients undergoing long-term topical therapy [39,40]. These findings suggest that the accumulation of specific medications in periocular tissues may provide alternative access to the posterior segment of the eye and the nearby vasculature.

As a result, topically applied ophthalmic medications have low bioavailability, requiring higher doses to achieve the desired effects. However, this increases the risk of side effects. Moreover, frequent daily dosing can be challenging and reduce patient adherence to treatment recommendations.

## 5. Limitations of Drug Formulations

**Figure 2:**
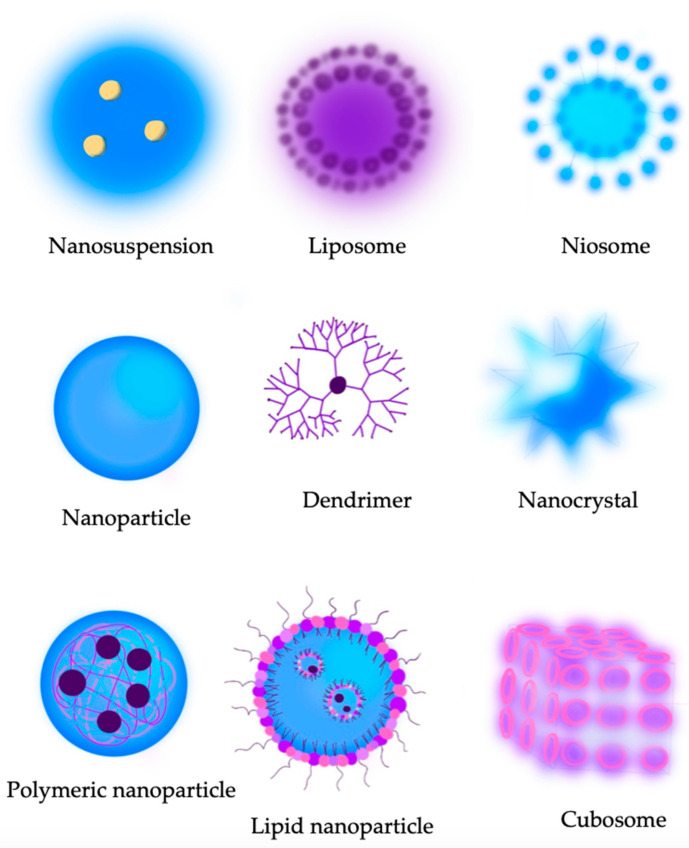
Types of nanocarriers that can be used in therapy for primary open-angle glaucoma.

There are many ways to deliver drugs to the eye. Conventional topical applications work well for diseases affecting the surface of the eye. However, for chronic diseases such as glaucoma, both doctors and patients still have problems with the precise dosage of a drug and difficulties associated with the daily usage of eye drops. These problems increase with the age of patients [41]. The bioavailability of instilled drugs is also significantly affected by the natural mechanisms of eye defense, such as the blinking reflex, lacrimation, or nasolacrimal drainage [42,43]. Another problem that may occur is the systematic toxicity of the drug. Nasolacrimal drainage and conjunctival blood vessels, combined with a release of the active substance that is too rapid, may lead to the achievement of doses that are harmless when administrated locally, but may cause side effects in a systemic context [44]. A breakthrough in the therapy of glaucoma, significantly facilitating drug administration and reducing the risk of errors in its application, may turn out to be ocular inserts, contact lenses, hydrogels, and nanoparticles (Figure 2).

## 6. Ocular Inserts

Ocular inserts (OIs) are synthetic medical devices containing drugs that are delivered to the surface of the eye using a sustained release mechanism [41,45]. They are extremely thin to reduce the unpleasant sensations that patients may experience, and they are also sterile, which is a critical requirement for ocular drugs. The selection of the synthetic medium is almost as important as the selection of the drug suspended in it. The main role of this administration of the drug is to significantly extend the contact time of the active substance with the conjunctival tissue. OIs are placed directly into the conjunctival sac. Inserts can be categorized as insoluble, in which the reservoir with the drug is located between polymers that release the substance in a controlled manner and match the type of substance. Another type, known as soluble inserts, represents the oldest variant. These inserts completely dissolve and do not require removal by the patient. The insert is placed in a solution containing the drug, and its amount will depend on the concentration of the solution, the soaking time, and the amount and type of drug binding agent. Lastly, bioerodible inserts comprise the third type, wherein drug release occurs gradually as the device comes into contact with tear fluid, eventually leading to its bioerosion.

The release of drugs can be achieved through three different mechanisms: diffusion, osmosis, and bioerosion [46]. In the process of diffusion, substances are continuously released into the tear fluid. When the membrane is non-eroding, the release occurs through microscopic pores. In soluble systems, the polymer swells when exposed to an aqueous solution, releasing the drug. The swelling is a result of the relaxation of the polymer chain. It is important for the patient to ensure adequate moisture on the eye surface, as dry membranes are impermeable to the drug. The dissolution time during the swelling process depends on the structure of the polymer, with the linear form dissolving faster than the cross-linked one. 

The construction of an OI is more complex [46]. It consists of a stretchable but insoluble membrane that divides the insert into two parts with different membrane permeabilities. When the insert comes into contact with the aqueous environment of the eye, the aqueous phase of the tear fluid diffuses into the first compartment, expanding it. Its expansion leads to the contraction of the second compartment, resulting in a release of the drug stored within it. 

In the treatment of glaucoma, a ring insert with bimatoprost is commonly used [47]. The ring is designed with a polypropylene core surrounded by an outer matrix composed of silicone. The structure ensures the stability and durability of the insert. Within this silicone matrix, 13 mg of bimatoprost is contained. The diameter of the ring insert is determined by the ophthalmologist based on the measurement of the patient’s intercanthal distance, allowing for optimal positioning and effectiveness of the ring insert.

Clinical trials comparing the use of inserts with typical eye drops have shown no significant difference in their IOP-reducing effects [48,49]. In both groups, the most frequently occurring side effect is mucoid discharge. Less common side effects include conjunctival hyperemia, punctate keratitis, and ocular pruritus. 

The use of OIs provides several benefits over eye drops [50]. One major advantage is the reduction in its frequency of administration. With a single correct application of the insert, the drug release is prolonged, with no need for frequent dosing throughout the day. This can be beneficial for older patients. The minimal loss of the administered drug during application is another advantage. The use of implants eliminates the need for preservatives, which are commonly found in traditional eye drops. By using preservatives-free inserts, the risk of sensitivity reactions or adverse side effects is reduced.

## 7. Contact Lenses

Contact lenses are curved plastic devices that are placed on the cornea and are typically used for the correction the ametropia conditions [49]. They can be tailored to individual patients based on their corneal shape, tear-film dynamics, and specific needs [50]. Contact lenses can be used for drug administration in addition to correcting refraction. Researchers have explored modifications of contact lenses to enable sustained drug release in the precorneal area [51]. This technique is based on the polymeric network of acrylate or silicone hydrogels with specific binding sites for incorporating drug molecules within the network. The bound drug is released into the post-lens tear fluid. Loading the drug into the contact lenses occurs through soaking, which leads to improved drug absorption in the cornea [52]. The drug residence time in the ocular tissue and tear film is increased. Drug-loaded contact lenses also help minimize drug loss through the nasolacrimal duct. Compared to conventional eye drops, contact lenses demonstrate higher efficiency in delivering drugs to the targeted ocular tissues and have the ability to correct refraction errors at the same time [53]. 

The water content in soft contact lenses plays a significant role in drug penetration [54]. Lower water content can reduce drug penetration due to decreased mobility of the drug molecules within lens matrices. Other crucial factors include the lens thickness and molecule sizes. Thinner lenses allow for faster drug diffusion through the materials, resulting in faster drug uptake. Thicker lenses provide a larger volume for drug incorporation and potentially allow for a longer release time. The size and structure of the drug molecule can affect its interaction with the polymeric network and its diffusion through the lens.

**Figure 3:**
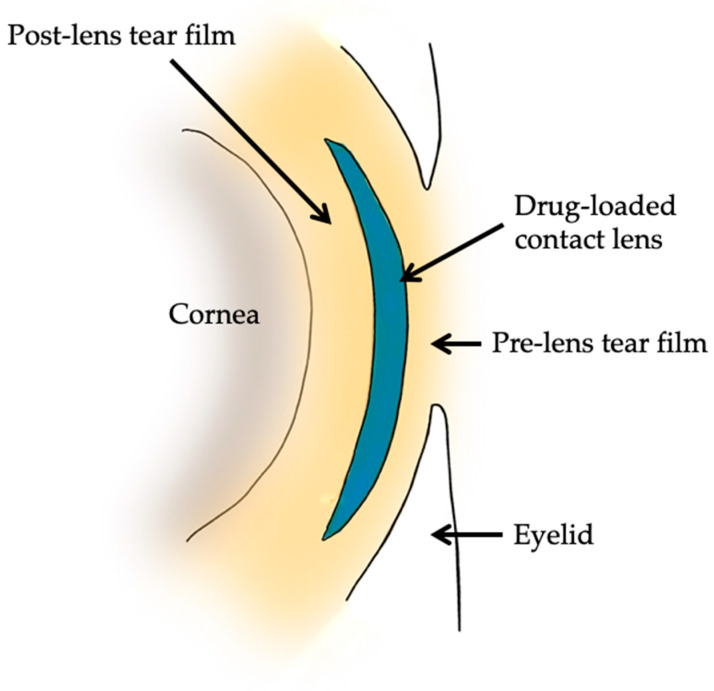
Schematic presentation of the application of drug-loaded contact lenses.

Innovative drug-loaded contact lenses for glaucoma treatment are currently still under development (Figure 3).

Table 1 presents a summary of the studies for glaucoma drug-loaded contact lenses and the differences between them (Table 1).

## 8. Hydrogels

Hydrogels are aqueous dispersions of hydrophilic polymers or copolymers that can form a three-dimensional structure capable of retaining large amounts of water or aqueous solutions [65]. They can be prepared in different forms, such as eye drops and contact lenses, and upon application, they undergo a transformation from a liquid to a gel-like consistency. This gelation process is triggered by factors like pH, temperature, or electrolyte concentration. 

In the context of ocular applications, hydrogels can be either in situ-formed or pre-formed [66]. In situ-formed hydrogels are created upon instillation into the eye and undergo a gelation process within the ocular environment. Pre-formed hydrogels are already in a gel state before application. Ion-activated polysaccharides are commonly used in hydrogel formulations. Polysaccharides in tears can form a gel-like hydrogel when crosslinked by the cations present in the tear fluid. This gel has increased viscosity, leading to prolonged drug retention, reduced drainage, and improved drug bioavailability in the eye [67]. The presence of cations in tears can further enhance the viscosity of the hydrogel. This extended contact time allows for once-daily administration and better adherence to treatment.

The use of hydrogels as drug carriers in glaucoma management has been investigated by researchers. Hydrogels offer several advantages, including their high water content, biocompatibility, and their ability to control drug release rates. Several polymers, such as chitosan, cellulose derivates, hyaluronic acid, gelatin, polyvinyl alcohol (PVA), and poly(lactic-co-glycolic acid) (PLGA), have been combined with hydrogels to enhance drug delivery efficacy [68,69,70,71,72].

## 9. Novel Technologies

### 9.1. Liposomes

Liposomes are unique spherical vesicles composed of phospholipid bilayers that enclose a watery interior [25,73]. They possess the ability to encapsulate and protect substances, making them highly valuable for drug delivery purposes. By modifying their composition and structure, liposomes can exhibit specific characteristics and respond to different stimuli, allowing for the controlled release of drugs. They can be classified into single-bilayer liposomes, including small unilamellar vesicles (SUVs) and large unilamellar vesicles (LUVs), as well as multilamellar vesicles (MLVs). These vesicles offer versatility in encapsulating both lipophilic and hydrophilic drugs, making them ideal for targeted therapies [74,75]. Liposomes have gained significant attention in ophthalmic treatments due to their biocompatibility, biodegradability, and lack of toxicity. They provide prolonged drug exposure on the ocular surface and are believed to have the potential to traverse the epithelium. Their small size enables them to pass through the corneal stroma, effectively reaching the front part of the eye.

Liposomes loaded with Latanoprost, using EggPC as the lipid component, exhibited a high drug-to-lipid ratio of 0.181 [76]. These liposomes demonstrated excellent stability over at least six months when stored at 4 °C and for at least one month at 25 °C. In vitro release studies showed a slow and sustained release profile, with 60% of the latanoprost released within 14 days. Furthermore, when compared to the daily administration of topical latanoprost, the same liposomal formulation displayed a significantly greater and sustained reduction in IOP beyond 90 days. The IOP lowering effect was measured to be approximately 4.8 mmHg compared to 2.5 mmHg for daily administration. Importantly, slit-lamp examination analysis revealed no signs of inflammation in the eyes, indicating the formulation’s safety and tolerability.

Research has indicated that iRGD-modified liposomes hold promise as a successful approach for ocular drug delivery [77]. Specifically, when brinzolamide, a medication used for glaucoma treatment, was loaded into iRGD-modified liposomes and applied topically, these liposomes were able to effectively penetrate the corneal barrier. This penetration occurred through a mechanism involving interaction with iRGD receptors, enabling enhanced therapeutic outcomes. The iRGD-modified liposomes demonstrated a more robust and prolonged therapeutic effect in treating glaucoma when compared to commercially available brinzolamide eye drops. These findings suggest that iRGD-modified liposomes could serve as an effective strategy for ocular drug delivery, offering improved treatment outcomes for glaucoma patients.

Two liposome formulations loaded with either brimonidine or travoprost were created using synthetic phospholipids [78]. These liposomes were enriched with compounds possessing osmoprotective activity. The designed formulations exhibited suitable physicochemical characteristics such as size, pH, osmolarity, surface tension, and viscosity, making them appropriate for administration to the ocular surface. They also demonstrated a high encapsulation efficiency, with brimonidine achieving an encapsulation efficiency of 24.78% and travoprost exhibiting an encapsulation efficiency of ≥99.01%. Furthermore, these liposomal formulations showed good tolerance in human corneal and conjunctival cell cultures, indicating their potential safety for ocular use. In addition, in vitro tests revealed their osmoprotective activity, suggesting a protective effect on the ocular surface. To evaluate the hypotensive effect, both liposomal formulations were tested in normotensive albino New Zealand rabbits. 

The results demonstrated that the liposomal formulations produced a faster and longer-lasting reduction in intraocular pressure compared to the corresponding commercialized products used as controls. Based on these findings, the combination of hypotensive liposomal formulations and osmoprotective agents holds great promise as a platform for the treatment of glaucoma while simultaneously protecting the ocular surface. This research opens up new possibilities for effective and comprehensive ocular therapies targeting both glaucoma and DED.

### 9.2. Niosomes

Niosomes are bilayer structures formed by non-ionic surfactants and have an amphiphilic nature. They range in size from 10 to 0.5 μm and are more stable than liposomes. Niosomes have the ability to encapsulate both hydrophilic and lipophilic active substances [79]. Spans^®^ and/or Tweens^®^ are the amphiphiles involved in niosomes production. The presence of surfactants allows them to permeate through the cornea, making them suitable for ocular delivery. They offer advantages such as a high formulation viscosity and excellent dispersion capacity on the corneal surface, leading to increased resistance to drainage. 

Niosomes have demonstrated improved drug bioavailability and positive therapeutic outcomes. Some studies have suggested that niosomes are more stable than liposomes [80]. 

Acetazolamide cannot be conventionally administered to the ocular surface due to its solubility characteristics. One study aimed to explore the topical delivery of acetazolamide using chitosan-STPP (sodium tripolophosphate) nanoparticles [81]. Niosomes loaded with acetazolamide have shown sustained drug release in the eye, following the Higuchi kinetic model. This drug release mechanism involved a combination of dissolution and diffusion. Acetazolamide-loaded niosomes resulted in a higher reduction in intraocular pressure (IOP) compared to plain drug solutions, with the effect lasting up to 5 h. Moreover, no signs of ocular irritation were observed. 

Niosome-in-gel systems for sustained ocular latanoprost delivery were also evaluated in vivo in rabbit eyes to assess their ability to reduce intraocular pressure [82]. The developed gel formulation was non-irritating and indicated nonspecific interactions between the latanoprost and various niosomal interactions. The result was a high drug encapsulation efficiency of 88% and sustained release of the drug from the niosomes.

In one study, the delivery of brimonidine-loaded niosomes resulted in a greater reduction in IOP compared to brimonidine-loaded liposomes [83]. The results of the niosome formulation demonstrated a 3 h longer IOP effect compared ton regular formulas, and the encapsulation efficiency of niosomes was higher than 32%. 

Niosomes loaded with timolol exhibited significantly higher maximum concentration (Cmax) and area under the curve (AUC) values in the aqueous humor compared to the timolol solution [84]. Niosomes facilitated efficient drug delivery through ocular barriers. The release of timolol was sustained, and the drug remained in the aqueous humor for an extended period. Timolol-loaded niosomes resulted in a prolonged reduction in IOP in rabbit eyes for eight hours, compared to two hours with the regular available solution. Niosomes have demonstrated prolonged drug release, enhanced bioavailability, and prolonged therapeutic effects. The use of bioadhesive coatings and specific formulations can further optimize drug delivery and efficacy while minimizing side effects.

### 9.3. Nanoemulsions 

Nanoemulsions are a type of emulsion with droplets size between 20 to 200 nanometers [85]. They are colloidal dispersions based on two immiscible liquids combined in the dispersed phase and continuous phase. In an oil-in-water nanoemulsion, the water is in a continuous phase, and the oil is dispersed. In a water-in-oil nanoemulsion, the oil phase serves as the continuous phase, and water is dispersed in it. In discontinuous nanoemulsions, both the aqueous and oil phases are interdispersed within the system, creating a continuous interfacial network between the two phases [86]. All formulations are composed of a selected surfactant and cosurfactants, facilitating a reduction in surface tension at the interface between the two immiscible phases. This leads to improved stability and performance of the nanoemulsion formulation by acting as penetration enhancer.

Nanoemulsions are one of the most preferred types of ocular drug delivery systems [87]. The small droplet size of nanoemulsions offers several advantages in terms of ingredient deposition and penetration into ocular barriers. The large surface area and low surface tension of the nanoemulsions improve the penetration efficacy of the ingredients. The solubility and permeability of many poorly soluble drugs can be enhanced by formulating them as nanoemulsions. Interestingly, nanoemulsions require lower amounts of surfactants during preparation, typically ranging from 3% to 10%, compared to microemulsions, which often necessitate surfactant concentrations of 20% or higher [88,89]. This lower surfactant requirement contributes to the fluidity and appealing physical properties of nanoemulsions, making them more desirable for topical applications.

The use of in situ gel-loaded nanoemulsion preparations has emerged as a promising alternative to commercial eye drops [87]. One key advantage of these preparations is their ability to provide a prolonged therapeutic effect. The sustained-release drug profile ensures a gradual release of the drug over time. This can result in less frequent application of the medication, improving patient compliance. The polymer forms a gel-like structure upon contact with the ocular environment, preventing the drug from being erased.

Catioprost^®^ is currently approved by the FDA as a nanoemulsion for the treatment of glaucoma and ocular hypertension [89]. It contains 0.005% latanoprost, and in vivo studies have shown a similar effectiveness to a reference product, Xalatan^®^, for lowering IOP. This preservative-free latanoprost 0.005% cationic emulsion was better tolerated, with no ocular irritation, compared to 0.02% BAK-preserved Xalatan in a rabbit model. Both Catioprost and its cationic emulsion formula have been shown to promote corneal healing and restore the function of the injured epithelium [90]. These results demonstrate the healing properties of nanoemulsions.

Morsi et al. investigated the use of acetazolamide nanoemulsion-based in situ gelling formulations [91]. They demonstrated a higher therapeutic efficacy compared to Azopt^®^ eye drops. Azopt is an ophthalmic suspension of brinzolamide. In particular, the acetazolamide in nanoemulsions formula exhibited the highest therapeutic activity, as evidenced by the higher AUC value (189.15 ± 10.18% h) compared to Azopt eye drops, with an AUC of 82.51 ± 7.53% h.

Previous studies have reported the successful formulation of nanoemulsions for the ocular delivery of pilocarpine, timolol, and dorzolamide [86,92].

### 9.4. Nanosuspensions and Nanocrystals

Nanosuspensions seem to be a promising and effective ocular delivery system for poorly water-soluble lipophilic drugs [93]. Nanosuspensions are a formulation consisting of submicron colloidal dispersions of drug nanocrystals surrounded by a stabilizer. The versatility of this drug delivery system can allow for integration with hydrogels by combining the characteristics of both systems. Nanocrystals are crystalline or partially crystalline forms with a diameter of less than 1 μm that have the ability to enhance drug solubility [94]. They can be incorporated into various drug delivery systems, such as nanosuspensions, liposomes, micelles, and hydrogels. Due to their large effective surface, they improve drug permeation through ocular barriers due to their ability to adhere to the junctions.

In situ gel-forming nanosuspensions have shown impressive results in drug delivery. A forskolin-loaded nanosuspension reduced IOP by 31% and maintained its efficacy for 12 h [95]. Brinzolamide nanocrystal formulations exhibited higher IOP-lowering efficacy (75% vs. 49% IOP reduction) than available brinzolamide drugs [96]. These studies highlight the promising advantages of using nanosuspensions with nanocrystal formulations as a drug delivery systems in the future.

El-Gendy et al. demonstrated the successful incorporation of an ocular penetration enhancer called Captex 8000 into liquid crystalline nanostructures for the delivery of Travoprost [97]. The nanocrystals showed improved penetration through the cornea and exhibited high stability and entrapment of the drug. The authors compared the commercially available drug Travatan^®^ with the new nanosuspension formula, showing a three-times higher bioavailability of the new drug delivery system. The pre-clinical studies were conducted in rabbits. These findings suggest that liquid crystalline nanostructures such as nanocrystals surrounded in stabilizer are promising therapeutic tools to enhance the effectiveness of anti-glaucoma management.

Betaxolol-loaded nanoparticles utilizing ion exchange resin (IER) complex suspensions have been approved by the FDA and are commercially available as Betoptic S^®^ [98]. This system contains 0.25% betaxolol, IER stabilizer, and polyacrylic polymer. It increases the residence time in the cul-de-sac of the eye. No significant difference was observed in terms of IOP reduction between 0.5% betaxolol solution eye drops and Betoptic S in a group of 352 patients with open-angle glaucoma. The ocular irritation seemed to be lower in the group using Betoptic S.

Donia et al. developed stable acetazolamide nanosuspensions with the use of stabilizers including poly-y-glutamic acid, hyaluronic acid, anionic polypeptide, and glycosaminoglycan [99]. The resulting formulation exhibited a uniform particle size distribution ranging from 100–300 nanometers and increased solubility of the drug. The dispersion characteristics were maintained for six months.

The use of nanocrystals in ocular drug delivery holds promise for improving therapeutic outcomes in the treatment of glaucoma. Their ability to enhance drug efficacy, provide sustained release, and minimize toxicity makes them an attractive option for developing novel ocular drug formulations.

### 9.5. Nanomicelles

Micelles are organized structures formed by amphiphilic molecules in aqueous media depending on the molecular weight of the hydrophilic and hydrophobic components [98]. The formation of micelles in water is ruled by several intermolecular forces. The critical micellar concentration is the concentration at which micelle formation occurs [99]. The CMS can be determined experimentally by measuring physical properties such as surface tension, conductivity, or osmotic pressure. 

Nanomicelles are micelles with a size below 100 nm and can be categorized into three types: surfactant nanomicelles, polymeric nanomicelles, and polyionic complex nanomicelles [100]. The hydrophobic core of nanomicelles can encapsulate hydrophobic drugs used to lower IOP, while the hydrophilic corona enhances their solubility and stability in aqueous environments. Surfactant nanomicelles are formed by low-molecular-weight surfactants like sodium dodecyl sulfate and tend to have high CMC values, indicating their limited stability. Polymeric micelles composed of amphiphilic copolymers have been developed to overcome these limitations.

Polymeric non-ionic surfactants are commonly used in ophthalmic delivery systems due to their advantages in terms of compatibility, stability, and toxicity [101]. They are generally less toxic, less hemolytic, and less irritating to the eye compared to cationic, anionic, or amphoteric polymers. One of their key benefits is an improvement in drug delivery to the ocular tissue. The small size and surfactant-like properties of nanomicelles allow them to penetrate through ocular barriers.

Pepić et al. used a micellar formulation of 2% poloxamer 407 to deliver pilocarpine [102]. Pilocarpine promotes the opening of the trabecular meshwork, increasing the drainage of aqueous humor in the treatment of glaucoma. The authors developed poloxamer 407 blank micelles with a size of 16.5 nm, 2% pilocarpine-loaded nanomicelles with a size of 18.7 nm, and 1.7% pilocarpine-loaded nanomicelles with a size of 30.3 nm. They implemented the drug into the outer shell of the micelles and in the hydrophobic core in the third formulation. In vivo experiments were conducted in albino rabbits by administering 25 µL of the micellar formulations or a 2% pilocarpine hydrochloride solution as a control. The miotic response duration in the rabbits was significantly prolonged with the micellar formulations compared to the control. The micelles containing pilocarpine hydrochloride extended the miotic response to approximately 180 min, while the micelles containing the pilocarpine base further extended the response to approximately 225 min.

The encapsulation of Dorzolamide resulted in a slight increase in micellar sizes [103]. The micelles were formed by copolymer A with a size of 39 nm and by copolymer B with a size of 47 nm. No interactions were found, and in vitro studies proved their non-toxic effects as well as their ability to sustain the release of a drug. The in vivo tests conducted on rabbit eyes confirmed the findings obtained from in vitro studies, demonstrating the efficacy of the designed micellar system in reducing intraocular pressure. 

The micellar system exhibited a long-lasting decrease in intraocular pressure, indicating its potential as an effective treatment option for glaucoma. The sustained release of drugs from the nanomicelles in the in vivo setting further supports their suitability for future in vivo tests and potential clinical applications. These findings suggest that they are safe and well-tolerated systems for drug delivery in the eye.

### 9.6. Polymeric Nanoparticles

Nanoparticles, specifically polymer-based nanocarriers, have been the most extensively studied for local ocular administration [104,105]. Based on their monomer properties, they are particles between 10 and 1000 nm and may have a surface charge that enhances the therapeutic’s mucoadhesion or permeability [105,106]. They can be classified as nanospheres or nanocapsules based on their internal structure [107,108]. Nanospheres are matrix systems in which the active ingredient is dispersed evenly within the polymer structure. Nanocapsules, on the other hand, consist of small deposits with an internal liquid core containing the active substance. The core is surrounded by a polymeric membrane, forming a reservoir system [109]. 

In a rabbit model, four types of ocular nanocarriers made from different polymers were tested to evaluate their effect on IOP reduction in the vitreous body. The polymers used included hyaluronic acid (HA), poly-L-lactic acid (PLLA), polystyrene (PS), and poly N-isopropylacrylamide (PNIPAM). Among these, PLLA, PNIPAM, and PS particles showed a significant reduction in IOP lasting for less than three days. The mean reduction in IOP was approximately 3, 4, and 6 mmHg (±2), respectively. The impact of HA particles on IOP reduction was minimal. The injection of these nanocarriers did not significantly affect the anatomical structure and thickness of retinal tissues, the cornea, and iris, except for PNIPAM particles, which caused statistically insignificant retinal thinning. 

One of the widely used polymera in ocular administration is PLGA. For glaucoma therapy, in vitro studies have investigated dendrimer–PLGA hybrid nanocarriers as carriers for brimonidine and timolol maleate [110]. These hybrid nanocarriers demonstrated sustained release of the drugs for 28–35 days. In vivo studies in rabbits with normal pressure showed that this formulation was capable of reducing IOP for four days after administration. In a glaucoma study conducted on rabbit corneas, the utilization of PLGA nanoparticles loaded with a combination of dexamethasone and melatonin resulted in a noteworthy reduction in IOP levels [68].

The hybrid nanocarriers enhanced the bioavailability of the active substances and maintained sustained IOP reduction, potentially reducing the frequency of daily administrations and improving therapeutic compliance. 

In 2018, Khan et al. developed chitosan-coated PLGA nanoparticles loaded with Forskolin [69]. The combination of PLGA (a synthetic polymer) and chitosan (a natural polymer) facilitated improved permeation and mucoadhesiveness on the surface of the cornea and sclera. The drug release study revealed a slow release of Forskolin nanoparticles, with 90% of the drug released over 72 h, in contrast to conventional drug suspensions, which released 96.6% within 12 h. Both polymers contributed to sustaining the release of the drug, resulting in a longer-lasting reduction in intraocular pressure (IOP).

Lee et al. conducted a study focused on glaucoma therapy in which they synthesized two types of nanoparticles to enable long-term and sustained release of pilocarpine [111]. Poly (ε-caprolactone) was employed to prepare nanocapsules (NCs) and nanospheres (NSs) as carriers for ocular drug delivery. The results indicated that the loading efficiency of pilocarpine in PCL NCs was significantly higher compared to PCL NSs, and the drug release followed a sustained pattern over time. Moreover, assessments of bioavailability, degradation rate, and in vivo experiments on rabbit eyes demonstrated that PCL NCs exhibited promising potential as carriers for glaucoma treatments. Notably, PCL NCs effectively reduced intraocular pressure in rabbit eyes, highlighting their therapeutic efficacy.

Abdel-Rashid et al. enhanced the ocular permeation and efficacy of acetazolamide employing chitosan, span 60, Tween^®^ 80/20, and sodium tripolyphosphate [112].

These findings highlight the potential of polymer-based nanocarriers as effective drug delivery systems for glaucoma treatment, offering sustained release and improved therapeutic outcomes. Further research is needed to optimize the formulations and evaluate their efficacy and safety in clinical settings.

### 9.7. Solid Lipid Nanoparticles

Lipid-based nanocarriers are emerging as a leading class of drug delivery systems. Among them, topical liposomal nanocarriers have gained significant attention in preclinical and early clinical studies for efficiently delivering ophthalmic solutions like timolol maleate to the vitreous and retina [113]. These lipid nanoparticles (NPs) are composed of oil-in-water emulsions, consisting of a lipid core and an amphiphilic surfactant as a stabilizer. Consequently, they can effectively transport both hydrophilic and hydrophobic drugs [114].

Lipid nanoparticles exhibit versatility, transitioning from a liquid to a solid state with various structures (e.g., steroids, monoglycerides, diglycerides, and triglycerides), and can be dispersed in aqueous solutions at ambient and body temperatures [115]. In the context of ophthalmic applications, lipid NPs are spherical vesicles composed of ionized lipids, exhibiting a positive charge at a neutral pH. Their size typically ranges from 40 to 1000 nm, and they are commonly classified as solid lipid nanoparticles (SLNs) and nanostructured lipid carriers (NLCs).

In a study conducted by Leonardi et al., cationic solid lipid nanoparticles (SLNs) loaded with melatonin were developed to enhance their hypotensive effect for the treatment of glaucoma [116]. The formulation utilized a cationic lipid to increase the electrostatic interaction between the cationic SLNs and the negatively charged mucin on the eye’s epithelial surface. This positive–negative charged interaction enhanced the mucoadhesive properties of the SLNs, leading to improved penetration and absorption of the drug. In vivo data demonstrated a reduction in IOP in albino rabbits for more than 24 h. The findings of the study indicated that SLNs offer promising potential as innovative carriers for the targeted delivery of anti-glaucomatous drugs.

Lipid NPs offer numerous advantages. They are commercially feasible and easily produced on a large scale. They are biocompatible and are well-tolerated by the body. Formulations containing lipid NPs with emulsifiers exhibit improved stability for both hydrophilic and lipophilic drugs, leading to controlled and extended drug retention in the body [114]. By reaching the lipid layer of the tear film, lipid NPs improve drug delivery and bioavailability upon topical instillation. Introducing positive surface charges through cationic lipids and surfactants further enhances the affinity of lipid NPs for lipid layers, extending their retention on the corneal epithelial layer. 

### 9.8. Nanostructured Lipid Carriers

Drugs incorporated into lipid nanoparticles are commonly used for surface application on the eye. Lipid nanoparticles, such as SLNs and NLCs, offer a promising platform for ocular drug delivery [117]. The lipid core that encapsulates the drug provides protection and controlled release. Lipid nanoparticles can adhere to the ocular surface and release the drug in a sustained manner, enhancing its therapeutic efficacy. Due to their lipophilic drug solubilizing effect and small particle size, lipid nanoparticles allow for better penetration into the eye.

Several recent studies have focused on evaluating the potential of nanostructured lipid carriers for ocular drug delivery in glaucoma patients. Chen et al. (2022) developed and evaluated nano-lipoidal carriers (NLCs) loaded with brinzolamide and latanoprost for the treatment of glaucoma [118]. The results showed that the size of the brinzolamide- and latanoprost-loaded NLCs was less than 200 nm, and the drug entrapment efficiency was high, at 97.5%. This suggests effective drug loading into the NLCs. Brinzolamide and latanoprost released from the NLCs exhibited significant permeation through the cornea. After 8 h, 50.5% and 49.4% of these drugs permeated the cornea, respectively. After 24%, the permeation increased to 81.4% and 84.2%, respectively, showing sustained drug release and a prolonged therapeutic effect. No toxicity or adverse effects were noted.

El-Salamouni et al. (2018) formulated and compared NLCs with SLSs and commercial eye drops for controlled delivery of brimonidine [119]. In terms of drug permeability, the NLCs demonstrated a permeability coefficient 1.227 times higher than SLNs, indicating improved corneal penetration. Histological studies revealed that NLCs reached the anterior ocular chamber, which was the target of the drug. The NLCs showed the most sustained and highest reduction in IOP, with a decrease of −13.14 ± 1.28 mmHg. This suggests that NLCs can effectively deliver brimonidine and enhance its ocular hypotensive effect compared to both commercial eye drops and SLNs.

### 9.9. Dendrimers

Dendrimers are polymers composed of repeating and regular branching units. Poly(aminoamine) (PAMAM) has been available commercially since 1989 [120]. Vandamme et al. evaluated the influence of PAMAM dendrimers on ocular drug delivery [121]. The authors investigated the in vivo residence time and ocular tolerance of different series of dendrimers in buffered phosphate solutions. The prolonged activities of dendrimer solutions of pilocarpine nitrate and tropicamide were examined in New Zealand albino rabbit eyes. The results showed that the presence of carboxylic and hydroxyl surface groups on the PAMAM dendrimers resulted in longer residence time in the eyes. However, increasing the concentration of dendrimers did not lead to further prolongation of the residence time. The remanence time on the cornea was found to be dependent on the size and molecular weight of the dendrimers. Additionally, the ocular retention time of PAMAM dendrimer solutions, fluorescein saline, and carbopol solutions were assessed. The PAMAM and carbopol solutions showed longer ocular residence periods compared to normal saline. Dendrimers can serve as effective ocular vehicles to enhance the residence time and improve ocular drug availability.

DenTimol, a dendrimer-based polymeric timolol analog, is a potential medication for glaucoma [122]. This study showed a high water solubility and no signs of toxicity or ocular irritation. A single topical application of DenTimol (10 μL of 0.5% *w/v* timolol) in normotensive adult Brown Norway male rats resulted in a significant reduction in intraocular pressure (IOP) in less than 30 min. The average IOP reduction was approximately 7.3 mmHg; a decrease of approximately 30%. This reduction in IOP was significantly higher compared to timolol phosphate-buffered saline (PBS) eye drops.

### 9.10. Cubosomes

Cubosomes hold promise as ophthalmic nanocarriers. They are a type of lipid-based nanocarrier composed of lipids organized in a bicontinuous cubic phase containing nanoparticles [123]. This unique structure consists of a network of water channels surrounded by lipid bilayers and provides a large surface area for drug loading, allowing for high drug encapsulation efficiency. They are analogous to other vesicular systems, such as liposomes and niosomes. Various techniques, including high-pressure homogenization, sonication, and solvent evaporation, have been employed to prepare cubosomes. They are able to accommodate all three types of molecules: hydrophobic, hydrophilic, and amphiphilic drugs within the lipid bilayers and the aqueous channels [124].

Del Valle Bessone et al. (2021) prepared latanoprost-loaded cubosomes using a top-down method in which the latanoprost concentration in the formulations varied from 0.00125% to 0.02% [125]. The results showed an average size of approximately 200 nm, and the encapsulation efficiency of latanoprost in the cubosomes was approximately 90%, indicating a high drug-loading capacity. The incorporation of the drug did not significantly alter the structure of the cubosomes. In vivo release studies demonstrated a slow and sustained release profile of latanoprost from the cubosomes over time. Researchers also evaluated the hypotensive intraocular effect in normotensive rabbits, with promising outcomes compared to widely available latanoprost formulations. This study suggests that cubosomes allow for sustained drug release and improved therapeutic efficacy.

Teba et al. (2021) developed different formulations of acetazolamide-loaded cubosomes [126]. They demonstrated enhanced corneal permeation and a superior reduction in IOP compared to commercially available eye drops (Azopt) and systemic tablets (Cidamex). The new formulation achieved a maximum decrease in IOP of 38.22%, while Azopt and Cidamex achieved decreases of 31.14% and 21.99%, respectively. The main residence time of the cubosomal formula was also prolonged.

The utilization of cubosomes holds promise for improving the ocular bioavailability and therapeutic efficacy of drugs, potentially offering a more effective and convenient treatment option for glaucoma patients.

### 9.11. Olaminosomes

Olaminosomes are new nanostructured platforms consisting of oleic acid, oleylamine, and sorbitan monooleate using the thin film hydration technique [127]. Oleic acid is an unsaturated free fatty acid, and oleylamine is a long-chain amino compound [128]. Their safety profile and biocompatibility make them attractive choices for implementation into nanocarriers. 

Only one research group has studied olaminosomes for ocular anti-glaucoma drug delivery. Abd-Elsalam et al. (2019) used olaminosomes to control agomelatine release in the eye [127]. Agomelatine has antidepressant features when administered orally and antiglaucomic features when administered topically to the eye [129]. To enhance the mucoadhesive properties of olaminosomes, the authors added chitosan HCL to the formulation. In vitro drug studies were used assess the release profile of agomelatine from the formulation and the reduction of IOP in rabbits after 8 h. The results were promising. Histopathological evaluation of rabbits’ eyes was performed to analyze any side effects or tissue damage, but the absence of changes suggested the safety of agomelatine-loaded olaminosomes. 

### 9.12. Bilosomes

Bilosomes are nanovesicles stabilized by bile salts and phospholipids [129]. Due to their resemblance to niosomes, they have been extensively studied for the vesicular carrier system. Bilosomes consist of nonionic surfactants and hyaluronic acid as an amphiphilic compound. These nanocarriers have shown promise as carriers for oral vaccines, transdermal delivery of tenoxicam, and ocular drug delivery [130,131].

Bilosomes have been explored for ocular drug delivery. Sakr et al. (2023) encapsulated betaxolol hydrochloride into highly permeable ocular bilosomes [131]. The aim of this study was to design betaxolol-loaded bilosomes for enhancing drug corneal permeability and prolonging their IOP-lowering effects. The new formulations revealed a 2.2-fold enhancement in betaxolol permeation compared to commercially available eye drops. No toxicity was found in histopathological studies. The betaxolol-loades bilosomes showed a superior IOP reduction (57.81%) compared to Epitaxol (35.27%). The overall results suggest the potential use of bilosomes for delivering hydrophilic molecules efficiently through the cornea.

Acetazolamide-loaded bilosomes were prepared using the thin film hydration method and various bile salts [132]. In vitro release studies revealed a biphasic release pattern evaluated in male albino New Zealand rabbits. This formulation improved and extended the lowering effect on IOP compared to plain acetazolamide, commercially available dorzolamide, and acetazolamide eye drops and tablets. This indicates the enhanced ocular delivery and sustained release from the bilosomal formulation (Table 2). 

## 10. Conclusions

Glaucoma is a significant global health concern that can result in visual impairment and blindness if left untreated. Early detection and management of the disease are crucial to prevent irreversible damage to the optic nerve. Lowering intraocular pressure, the main modifiable risk factor, is the primary goal of treatment. Pharmacological interventions, including prostaglandins, β-adrenergic blockers, carbonic anhydrase inhibitors, α-adrenergic agonists, and miotics are commonly used to reduce intraocular pressure and slow down disease progression. Surgical options such as laser therapy and implantation of stents provide additional avenues for managing glaucoma. 

Topically applied ophthalmic medications have a low bioavailability, requiring higher doses to achieve the desired therapeutic effects. However, higher dosages increase the risk of potential side effects. The need for frequent daily dosing poses challenges for patients and may lead to decreased adherence to treatment recommendations. Finding strategies to improve the bioavailability of ophthalmic medications and exploring alternative drug delivery methods are important areas of research. 

Nanotechnology holds significant promise in glaucoma treatment by revolutionizing drug delivery, enhancing therapeutic outcomes, and improving patient quality of life. One of the most promising nanotechnologies in glaucoma treatment is the development of nanoparticle-based drug delivery systems. Nanoparticles can encapsulate glaucoma medications, protecting them from degradation and extending their release over time. This controlled release allows for sustained drug delivery, reducing the need for frequent eye drops, potential side effects, and improving patient compliance. While challenges remain, including regulatory approval and clinical translation, ongoing research in nanotechnology-driven approaches is likely to contribute to more effective and targeted glaucoma therapies in the future.

## Figures and Tables

**Table 1:** Summary of the studies for glaucoma drug-loaded contact lenses.

Authors	Materials	Fabrication Method	Drug-Loaded	Drug Loading Technique	Results
Mohamdeen et al. (2022) [55]	Polylactic acid (PLA)	3D printing	Timolol maleate	Drug solution added to polymeric formulation	Sustained release of timolol maleate for 3 days
Li et al. (2021) [56]	Gold nanoparticles	Synthesis of gold nanoparticles through the reduction of chloroauric acid	Bimatoprost	Drug solution loaded into nanoparticles and nanoparticles implemented into contact lense matrix	Sustained bimatoprost release up to 72 h
Hsu et al. (2015) [57]	Vitamin E	Incorporation of vitamin E	Timolol and dorzolamide	Vitamin E-loaded lenses for timolol and dorzolamide loaded separately and then together in the same lens	Increased the release durations of both drugs to 2 days
Maulvi et al. (2016) [58]	Cellulose nanoparticles	Synthesis of ethyl cellulose nanoparticles	Timolol maleate	Soaking	Reduced IOP for eight days in a rabbit glaucoma model
C de Guzman et al. (2022) [59]	Silicone	Polymerization of monomers	Brinzolamide	Soaking	Enhanced drug loading capacity and release
Ciolino et al. (2014) [60]	PLGA	Encapsulating contact lenses with PLGA-Latanoprost film	Latanoprost	Drug-loaded platform embedded in hydrogel-based lenses	Sustained release of latanoprost for 1 month
Kim et al. (2014) [61]	Diamond particles	Lysozyme-dependent polysaccharide degradation-mediated diamond nanogel contact lenses development	Timolol maleate	Soaking	Enhanced drug loading capacity and release
Sekar et al. (2019) [62]	Vitamin E	Incorporation of vitamin E into ACUVUE^®^OASYS^®^ and ACUVUE^®^ TruEyeTM	Bimatoprost and latanoprost	Soaking	Increased the release durations of both drugs to 10 days
Wei et al. (2021) [63]	Microemulsion	Microemulsion soaking emulsion	Timolol	Imprinting and soaking	Enhanced drug loading capacity and release to 96 h
Ciolino et al. (2016) [64]	Methafilicon and methacrylic acid	Photopolymerization	Latanoprost	Soaking	

**Table 2:** Advantages and disadvantages of the described nanotechnologies for glaucoma treatment.

Novel Technology	Possible Drugs Loaded	Advantages of the Technology	Disadvantages of the Technology
Liposomes	Latanoprost, brinzolamide, travoprost	Biocompatibility, biodegradability, lack of toxicity, prolonged drug presence on the ocular surface, potential to traverse the epithelium, reach the eye by effectively penetrating through the corneal stroma due to the small size, safety for the patients, a faster and longer-lasting reduction in intraocular pressure compared to other products, prolonged half-life of a drug	Might cause blurring after injection to the vitreous humour, limited storage conditions
Niosomes	Acetazolamide, brimonidine, timolol,	High formulation viscosity, excellent dispersion capacity on the corneal surface, increased resistance to drainage, low toxicity, non-immunogenic, ability to encapsulate hydrophilic and lipophilic active substances	Physical instability, aggregation, inefficient drug loading
Nanoemulsions	Acetazolamide, latanoprost, pilocarpine, dorzolamide, timolol	Good stability and penetatration throught the corneal surface, enhance the solubility and permeability of many poorly soluble drugs, controlled drug release	Lower permeability and drug bioavailability
Nanosuspensions and nanocrystals	Forskolin, brinzolamide, travoprost, betaxolol, acetazolamide	Enhanced drug solubility, enhanced stability, improved drug permeation through ocular barriers, high drug loading, increased bioavailability	Accurate doses cannot be achieved unless suspended
Nanomicelles	Pilocarpine, dorzolamide	Lower toxicity, small particle size, high drug loading and water solubility, good structurla stability, protects drugs from environemntal conditions, controlled drug release	Occasional cytotoxicity, sometimes surface modifications are needed, low drug-loading capacity, can only be used for lipoholic drugs
Polymeric nanoparticles	Brimonidine, timolol, a combination of dexamethasone and melatonin, forskolin, acetazolamide	Can be used for both hydrophilic and hydrophobic drugs, high stability, controlled drug release	Insufficient data regarding toxicity
Solid lipid nanoparticles	Melatonin	Can be used for both hydrophilic and hydrophobic drugs, biocompatibile and well tolerated by the body, easily produced on a larger scale, controlled and extended drug retention in the body, long stability, lower cytotoxicity, improves drug bioavailability	Insufficient amount of clinical studies, drug expulsion during storage
Nanostructured lipid carriers	Brinzolamide, latanoprost, brimonidine	Higher drug loading capacity, low toxicity and cytotoxicity	Low drug loading and risk of gelation for SLNs
Dendrimers	Pilocarpine, tropicamide, timolol	Controlled pharmacokinetics, increased drug solubility, stability, and permeability, low toxicity, non-immunogenic, improved bioavailability, high purity and uniformity, controlled drug release	Inssuficient amount of clinical studies, difficulties in synthesizing large quantities pure enough to be used in clinical trials
Cubosomes	Latanoprost, acetazolamide	Ability to accommodate hydrophobic, hydrophilic, and amphiphilic drugs, biocompatibile, low toxicity, good thermodynamic stability	Large-scale production might be difficult due to the high viscosity
Olaminosomes	Agomelatine	Good safety profile, biocompatibility	Insufficient amount of research
Bilosomes	Acetazolamide, betaxolol hydrochloride	Ability to deliver hydrophilic molecules	Insufficient amount of research

## Abbreviations

ACZ	Acetazolamide
BAK	Benzalkonium chloride
COPD	Chronic obstructive pulmonary disease
EDTA	Ethylenediaminetetraacetic acid
IOP	Increased intraocular pressure
LUVs	Large unilamellar vesicles
MLVs	Multilamellar vesicles
NCs	Nanocapsules
NLCs	Nanostructured lipid carriers
NPs	Nanoparticles
NSs	Nanospheres
OIs	Ocular inserts
OMDI	Omidenepag isopropyl
PBS	Phosphate-buffered saline
PLGA	Poly(lactic-co- glycolic acid)
PVA	Polyvinyl alcohol
SLNs	Solid lipid nanoparticles
SUVs	Small unilamellar vesicles
WHO	World Health Organization
